# Growth arrest and DNA damage 45γ is required for caspase-dependent renal tubular cell apoptosis

**DOI:** 10.1371/journal.pone.0212818

**Published:** 2019-02-22

**Authors:** Gyu-Tae Shin, Hwa Joung Lee, Ji Eun Park

**Affiliations:** Department of Nephrology, Ajou University School of Medicine, Suwon, Korea; National Institutes of Health, UNITED STATES

## Abstract

**Background:**

Growth Arrest and DNA Damage 45γ (GADD45γ) is a member of the DNA damage-inducible gene family which responds to environmental stresses. Apoptosis is a critical mode of renal tubular cell death in nephrotoxin-induced acute kidney injury. In this study, we investigated the role of GADD45γ in renal tubular cell apoptosis induced by nephrotoxic drugs.

**Methods:**

Primary human renal tubular epithelial (HRE) cells were used in this study. To derive stable cell lines in which GADD45γ expression was silenced, HRE cells were transduced with a plasmid encoding GADD45γ-specific shRNA. The recombinant adenovirus containing the GADD45γ gene was synthesized to overexpress GADD45γ protein. Cell death was induced by cisplatin and cyclosporine A (CsA). To prevent apoptotic cell death, pan-caspase inhibitor ZVAD-FMK was used. To prevent non-apoptotic cell death, necrostatin-1 and ferrostatin-1 were used. The degree of apoptosis and necrosis of cultured cells were evaluated by flow cytometry.

**Results:**

Expression of the GADD45γ gene was significantly upregulated in response to treatment with CsA and cisplatin. Apoptosis and necrosis induced by these drugs were significantly reduced by silencing of GADD45γ, and significantly augmented by the overexpression of GADD45γ. The activation of caspase-3 and caspase-7 as well as caspase-9 induced by cisplatin or CsA was reduced by silencing of GADD45γ, and was augmented by the overexpression of GADD45γ, indicating that caspase activation is dependent on the expression of GADD45γ. ZVAD-FMK significantly inhibited apoptosis induced by cisplatin or CsA, indicating a role of caspases in mediating apoptotic cell death. ZVAD-FMK was effective to prevent necrosis as well, indicating that the observed necrosis was a secondary event following apoptosis at least in part.

**Conclusions:**

To our knowledge, this is the first study to show that GADD45γ is required for the caspase-dependent apoptosis of renal tubular cells induced by nephrotoxic drugs.

## Introduction

Growth Arrest and DNA Damage 45γ (GADD45γ), an isoform of the GADD45 family of proteins, is a molecule which responses to environmental stresses by checking on the cell cycle [1], and by inducing apoptosis [2]. Apoptosis is a critical mode of renal tubular cell death in acute kidney injury (AKI) and prevention of apoptosis was shown to protect renal function [3]. With regard to kidney damage, we previously showed that GADD45γ contributes to the progression of chronic kidney disease in a mouse model of chronic tubular injury [4] and human chronic glomerulonephritis [5]. To date, however, no data exists with regard to the role of GADD45γ in AKI, prompting us to investigate its role in apoptosis of renal tubular cells. Tubular cell death in AKI resulting from direct renal insults such as renal ischemia [6, 7], sepsis [8], and nephrotoxins [9–13] was shown to proceed through apoptosis. For our *in vitro* experiments, we selected the nephrotoxic drugs cisplatin and cyclosporine A (CsA) to evaluate the link between GADD45γ and renal tubular cell apoptosis. Cisplatin is a widely used chemotherapy drug, but its use is limited by its nephrotoxicity [14]. Nephrotoxicity by cisplatin involves necrosis as well as apoptosis of renal tubular cells, and the suppression of apoptosis has been shown to be protective against cisplatin-induced renal injury [10]. CsA was the first approved calcineurin inhibitor and has been extensively used in kidney transplantation to prevent acute rejection. However, ironically, CsA causes kidney injury [15, 16], and nephropathy caused by CsA has been associated with a marked increase in apoptosis of tubular and interstitial cells [17].

Through a series of experiments, we have found convincing evidence that GADD45γ is indispensable for the activation of caspases, and caspase-mediated renal tubular cell apoptosis is determined by the level of GADD45γ expression. In this paper, we present novel findings that implicate GADD45γ in the nephrotoxin-induced apoptotic pathways of renal tubular cells.

## Materials and methods

### Primary human renal tubular epithelial (HRE) cell culture

HRE cells were purchased from Lonza (Walkersville, MD) and were maintained in Renal Epithelial Basal Medium supplemented with 10% FBS and the SingleQuots kit (Lonza).

### Construction of GADD45γ knockdown HRE cell lines

To knockdown GADD45γ expression in HRE cells, we used the vector containing short hairpin RNA (shRNA) composed of the target sequence CGTCTACGAGTCAGCCAAAGT, the loop CTTCCTGTCA, the U1 promoter, and puromycin resistance gene (SA bioscience, Frederick MD). The negative control (NC) vector contained the insert sequence GGAATCTCATTCGATGCATAC which has no homology to known gene sequences. HRE cells were transfected with each vector using SureFECT transfection reagent (SA bioscience) and the cells were selected using 3 ug/ml puromycin (Invivogen, San Diego, CA) to generate stable cell lines expressing the shRNA constructs that target the GADD45γ (shRNA-GADD45γ), or no known genes (shRNA-NC).

### Construction of recombinant adenoviruses expressing GADD45γ

The complete open reading frame of human GADD45γ in pENTR221 (Invitrogen, Carlsbad, CA) was transferred to the pAd/CMV/V5-DEST vector (Invitrogen) by LR recombination. After sequencing to confirm the orientation and positions, the plasmids were transfected into HEK293A cells to produce recombinant adenoviruses. For controls, recombinant adenoviruses containing the lacZ gene were produced. For purification and concentration, the Adeno-X maxi purification kit (Clontech, Mountain View, CA) was used. For titration, HEK293 cells infected with recombinant adenoviruses were detected using an antibody specific for the adenovirus hexon protein with the Adeno-X rapid titer kit (Clontech). HRE cells were infected with adenoviral vectors harboring the GADD45γ gene or LacZ gene for 48 hours with a multiplicity of infection (MOI) of 25–50 before treatment with cisplatin or CsA.

### Cell death induction

To induce cell death, HRE cells were treated with cisplatin intravenous solution (0.5 mg/ml in normal saline, Ildong pharmaceuticals, Seoul, Korea), or cyclosporine A. Cyclosporine A (Sigma-Aldrich, St. Louis, MO) was dissolved in ethanol to yield a 10 mg/ml stock. Pan-caspase inhibitor ZVAD-FMK (R&D Systems, Minneapolis, MN) was dissolved in DMSO to yield a 20 mM stock solution. Necrostatin-1 (Sigma-Aldrich), an inhibitor of necroptosis, was dissolved in DMSO to yield a 38.5 mM stock solution. Ferrostatin-1 (Sigma-Aldrich), an inhibitor of ferroptosis, was dissolved in DMSO to yield a 38.1 mM stock solution. The reagents were used at the following concentrations: cisplatin (12.5–200 μM), cyclosporine A (25 ug/ml), ZVAD-FMK (25 μM), necrostatin-1 (50 μM), ferrostatin-1 (1 μM).

### RNA isolation

Samples were homogenized in Trizol reagent (Invitrogen). RNA was extracted with chloroform, precipitated with isopropanol, washed with 75% ethanol, and then re-dissolved in TE buffer. The isolated RNA was quantified spectrophotometrically.

### Reverse transcription-polymerase chain reactions (RT-PCR)

After removing contaminating DNA from the isolated RNA using DNase I (Invitrogen), 2–3 μg of total RNA was reverse transcribed into cDNA in the reaction mixtures containing Moloney murine leukemia virus reverse transcriptase and random hexanucleotide primers. The individual PCR was performed for 28–35 cycles. The amplification profile typically consisted of denaturing at 94°C for 20 seconds, primer annealing at 57°C for 30 seconds and primer extension at 72°C for 20 seconds. Primers used are listed in Table 1.

**Table 1:** Primers used in the polymerase chain reactions.

Primers expected size	Sequences	GenBank Accession
GADD45γ 300bp	F: 5'-AAGCGCTGCATGAGTTGCTG-3' R: 5'-TCGAAATGAGGATGCAGTGCA-3'	NM_006705
GAPDH 299bp	F: 5'-GTCGGAGTCAACGGATTTGG-3' R: 5'-ATGGTGGTGAAGACGCCAGT-3'	NM_002046

F: forward primers, R: reverse primers, GADD45γ: Growth Arrest and DNA Damage 45γ, GAPDH: glyceraldehyde-3-phosphate dehydrogenase

### Protein extraction

Cultured cell monolayers were scraped in lysis solution (Cell signaling, Danvers, MA) containing protease inhibitors (Protease Inhibitor Cocktail, Roche Life Science, Indianapolis, IN) and phosphatase inhibitors (PhosStop, Roche Life Science). The homogenates were incubated on ice for 30 minutes, and then centrifuged to pellet cell debris at 13,000 rpm for 10 minutes at 4°C. Protein was quantified using the Bradford dye-binding method (Bio-Rad, Hercules, CA).

### Western blot analysis

Equal amounts of total protein (10–20 μg) were subjected to 10 to 15% acrylamide gel SDS-PAGE, transferred to a polyvinylidene difluoride (PVDF) membrane, and then incubated overnight at 4°C with primary caspase antibodies (Cell signaling) and a phospho-mixed lineage kinase domain-like **(**MLKL) antibody (Cell signaling). The membrane was incubated with peroxidase-conjugated secondary antibodies for 1 hour, and the proteins were visualized by enhanced chemiluminescence (Amersham, Piscataway, NJ).

### Detection of apoptosis and necrosis by flow cytometry

Cells were harvested by trypsinization, pooled with the culture medium containing floating cells, collected by centrifugation, and incubated with the Annexin V-FITC conjugate and propidium iodide (PI) for 10 minutes using an apoptosis detection kit (APOAF, Sigma-Aldrich), then analyzed by flow cytometry. The percentage of apoptosis and necrosis was evaluated by plotting cell staining by Annexin V (x-axis) vs PI (y-axis). The upper left quadrant (PI positive and Annexin V negative, Q1) represents necrotic/non-viable cells; the upper right quadrant (PI positive and Annexin V positive, Q2) represents necrotic/late apoptotic cells; the lower left quadrant (PI and Annexin V negative, Q3) represents live cells; and the lower right quadrant (PI negative and Annexin V positive, Q4) represents early apoptosis.

### Statistical analysis

Data were expressed as mean ± SD. Student’s t-test or one-way ANOVA followed by Bonferroni's post-hoc comparisons tests were used to compare the groups. A p value <0.05 was considered significant.

## Results

### GADD45γ mRNA expression is induced by CsA and cisplatin in renal tubular cells

**Fig 1:**
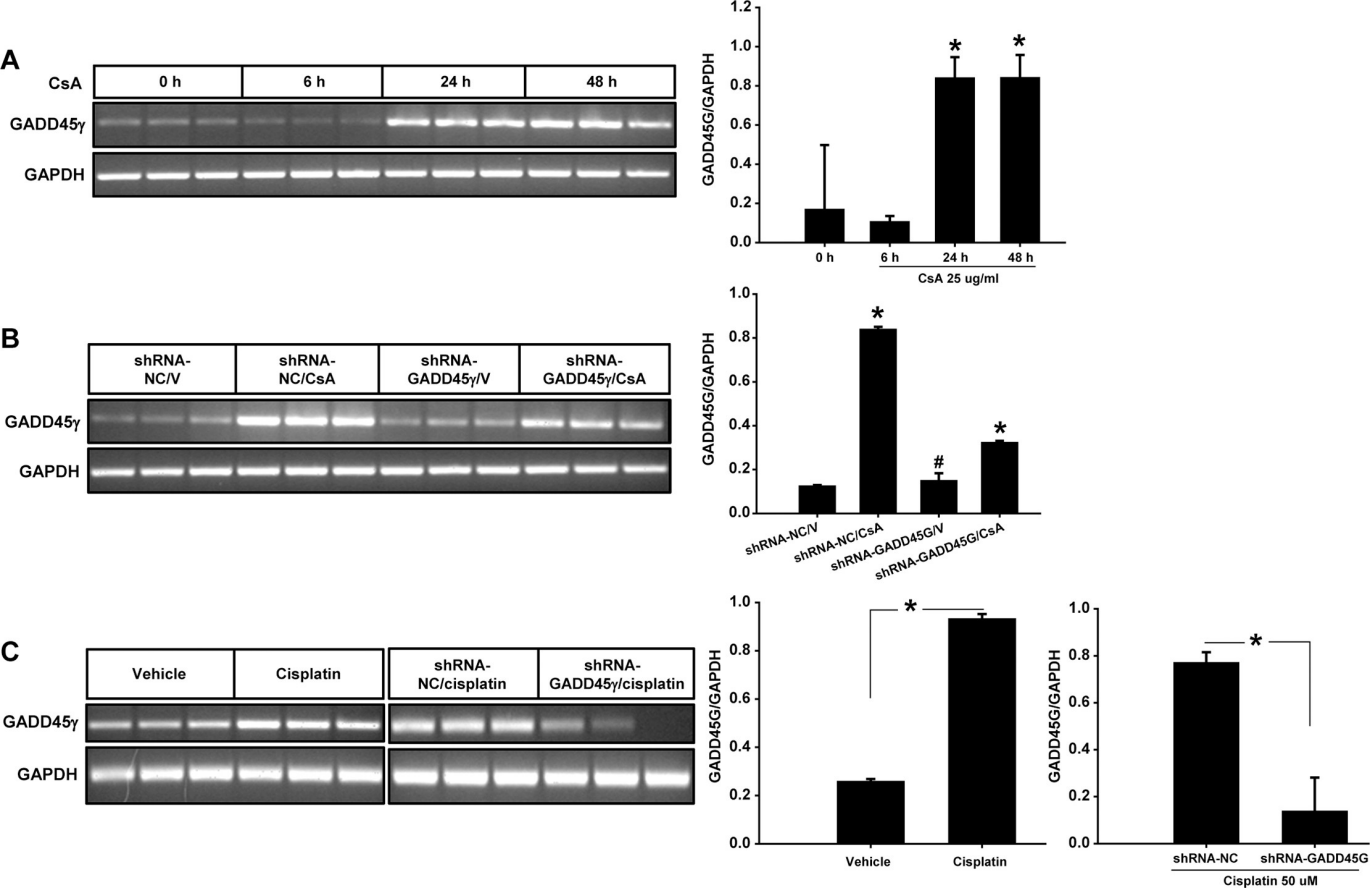
RT-PCR for mRNA expression in CsA- and cisplatin-treated renal tubular cells. HRE cells were incubated with CsA or cisplatin to measure GADD45γ mRNA expression using RT-PCR. GAPDH served as an internal control. **(A)** The time course of the CsA-induced gene expression of GADD45γ. HRE cells were incubated with 25 ug/ml CsA for the indicated time. *p<0.05 vs 0 hours and 6 hours. **(B)** shRNA-GADD45γ and shRNA-NC stable HRE cell lines were incubated with 25 ug/ml CsA for 24 hours. V represents vehicle. *p<0.05 vs all other values. #p<0.05 vs shRNA-NC/CsA and shRNA-GADD45γ/CsA. **(C)** HRE cells were incubated with 25 μM cisplatin for 12 hours (left panel), and shRNA-GADD45γ and shRNA-NC stable HRE cell lines were incubated with 25 μM cisplatin for 18 hours (right panel). *p<0.05 between the groups. shRNA-GADD45γ represents shRNA constructs to knockdown GADD45γ; shRNA-NC, shRNA constructs targeting no known genes. Data were expressed as mean ± SD. Student’s t-test (panel C) or one-way ANOVA followed by Bonferroni's post-hoc comparisons tests (Panel A and B) were used to compare the groups. A p value <0.05 was considered significant.

HRE cells were incubated with CsA or cisplatin and the expression of mRNA was evaluated by RT-PCR. GADD45γ mRNA was induced within 24 hours of CsA treatment (Fig 1A), and the induction was effectively blunted by shRNA-GADD45γ expression (Fig 1B). Similarly, GADD45γ mRNA was significantly induced by cisplatin treatment, and the induction was effectively blunted by shRNA-GADD45γ expression (Fig 1C).

### Cisplatin-induced renal tubular cell death is dependent on GADD45γ expression

**Fig 2:**
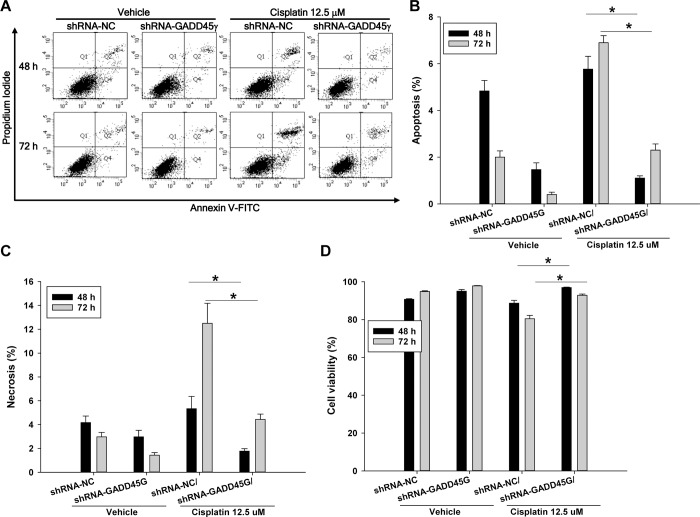
Quantitation of apoptosis and necrosis by flow cytometry in cisplatin (12.5 μM)-treated renal tubular cells: The effect of GADD45γ knockdown. shRNA-GADD45γ and shRNA-NC stable HRE cell lines were treated with 12.5 μM cisplatin for indicated time periods. **(A)** Representative scatter plots of flow cytometry. Quantitation of **(B)** apoptotic cells (PI negative and Annexin V positive, Q4), **(C)** necrotic cells (PI positive and Annexin V positive, Q2), **(D)** viable cells (PI negative and Annexin V negative, Q3). Each bar represents mean ± SD of 3 experiments. *p < 0.05 (One-way ANOVA followed by Bonferroni's post-hoc comparisons tests). shRNA-GADD45γ(G) represents shRNA constructs to knockdown GADD45γ; shRNA-NC, shRNA constructs targeting no known genes.

**Fig 3:**
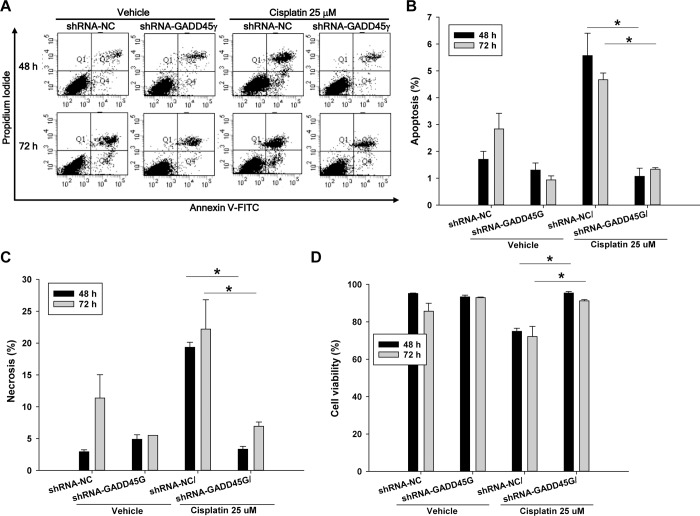
Quantitation of apoptosis and necrosis by flow cytometry in cisplatin (25 μM)-treated renal tubular cells: The effect of GADD45γ knockdown. shRNA-GADD45γ and shRNA-NC stable HRE cells lines were treated with 25 μM cisplatin for indicated time periods. **(A)** Representative scatter plots of flow cytometry. Quantitation of **(B)** apoptotic cells (PI negative and Annexin V positive, Q4), **(C)** necrotic cells (PI positive and Annexin V positive, Q2), **(D)** viable cells (PI negative and Annexin V negative, Q3). Each bar represents mean ± SD of 3 experiments. *p < 0.05 (One-way ANOVA followed by Bonferroni's post-hoc comparisons tests). shRNA-GADD45γ(G) represents shRNA constructs to knockdown GADD45γ; shRNA-NC, shRNA constructs targeting no known genes.

**Fig 4:**
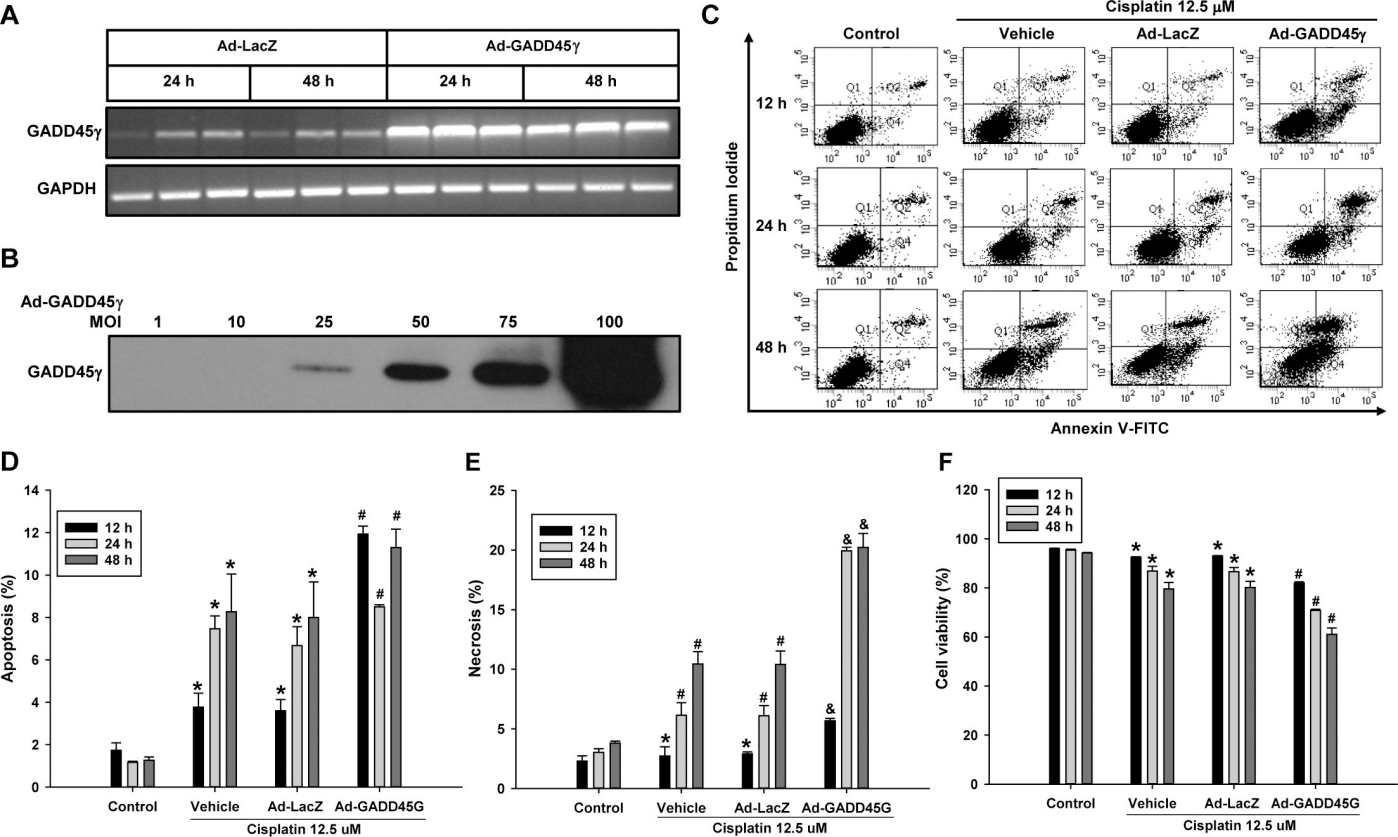
Quantitation of apoptosis and necrosis by flow cytometry in cisplatin-treated renal tubular cells: The effect of GADD45γ overexpression. **(A)** Detection of overexpressed GADD45γ RNA by RT-PCR. HRE cells were infected with 50 MOI of adenoviral vectors for 24–48 hours. **(B)** Detection of overexpressed GADD45γ protein by Western blot. HRE cells were infected with a serial amount of Ad-GADD45γ for 48 hours. HRE cells were infected with 50 MOI of adenoviral vectors for 48 hours and then treated with 12.5 μM cisplatin for indicated time periods (panels C-F). **(C)** Representative scatter plots of flow cytometry. **(D)** Quantitation of apoptotic cells (PI negative and Annexin V positive, Q4). *p<0.05 vs control and Ad-GADD45γ/cisplatin. #p<0.05 vs all other corresponding values. **(E)** Quantitation of necrotic cells (PI positive and Annexin V positive, Q2). *p<0.05 vs Ad-GADD45γ/cisplatin. #p<0.05 vs control and Ad-GADD45γ/cisplatin. &p<0.05 vs all other corresponding values. **(F)** Quantitation of viable cells (PI negative and Annexin V negative, Q3). *p<0.05 vs control and Ad-GADD45γ/cisplatin. #p<0.05 vs all other corresponding values. Each bar represents mean ± SD of 3 experiments. *p < 0.05 vs corresponding Ad-LacZ transduced cells (One-way ANOVA followed by Bonferroni's post-hoc comparisons tests). MOI, multiplicity of infection. Ad-GADD45γ(G) represents adenoviral vectors expressing GADD45γ; Ad-LacZ, LacZ adenoviral vectors.

To investigate the role of GADD45γ in cisplatin-induced cell death, GADD45γ knockdown shRNA-GADD45γ HRE cells were incubated with 12.5 μM cisplatin and then analyzed by flow cytometry. Apoptosis as well necrosis was significantly decreased by silencing of GADD45γ, and led to significantly increased survival of shRNA-GADD45γ stable cells, compared to negative control shRNA-NC stable cells (Fig 2). Similar results were obtained when the cells were incubated with a higher concentration of cisplatin, 25 μM (Fig 3). To further clarify the role of GADD45γ in cell death, HRE cells overexpressing GADD45γ were made using adenoviral vectors, incubated with 12.5 μM cisplatin, and analyzed by flow cytometry. In accordance with GADD45γ knockdown experiments, apoptosis as well as necrosis was significantly increased in GADD45γ- overexpressing cells compared to LacZ control cells during the cisplatin incubation time, resulting in significantly decreased viability of GADD45γ-overexpressing cells (Fig 4). This data indicates that GADD45γ play a key role in controlling cisplatin-induced cell death in renal tubular cells.

### CsA-induced renal tubular cell death is dependent on GADD45γ expression

**Fig 5:**
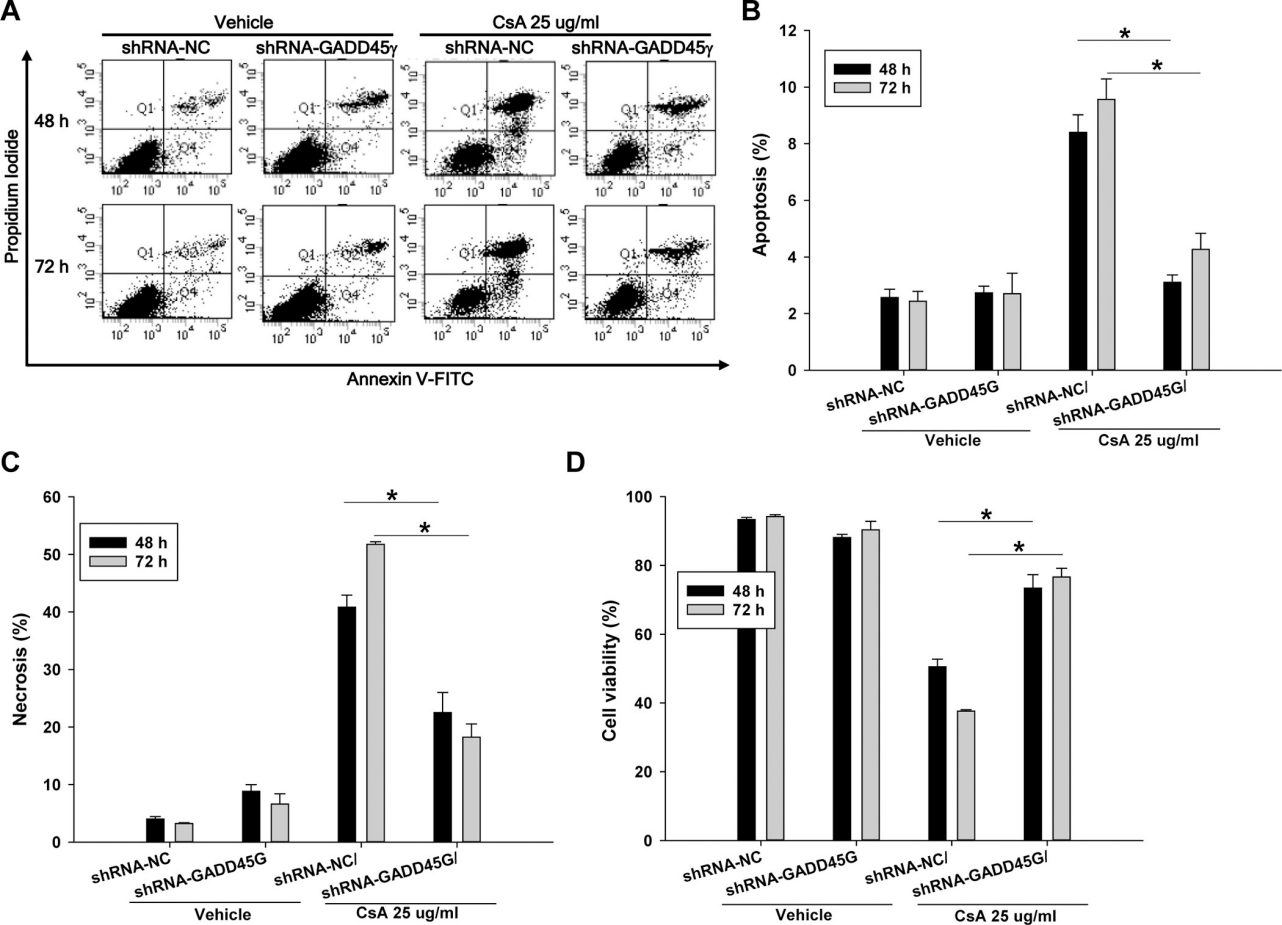
Quantitation of apoptosis and necrosis by flow cytometry in CsA-treated renal tubular cells: The effect of GADD45γ knockdown. shRNA-GADD45γ and shRNA-NC stable HRE cells lines were treated with 25 μg/ml CsA for indicated time periods. **(A)** Representative scatter plots of flow cytometry. Quantitation of **(B)** apoptotic cells (PI negative and Annexin V positive, Q4), **(C)** necrotic cells (PI positive and Annexin V positive, Q2), **(D)** viable cells (PI negative and Annexin V negative, Q3). Each bar represents mean ± SD of 3 experiments. *p < 0.05 (One-way ANOVA followed by Bonferroni's post-hoc comparisons tests). shRNA-GADD45γ(G) represents shRNA constructs to knockdown GADD45γ; shRNA-NC, shRNA constructs targeting no known genes.

**Fig 6:**
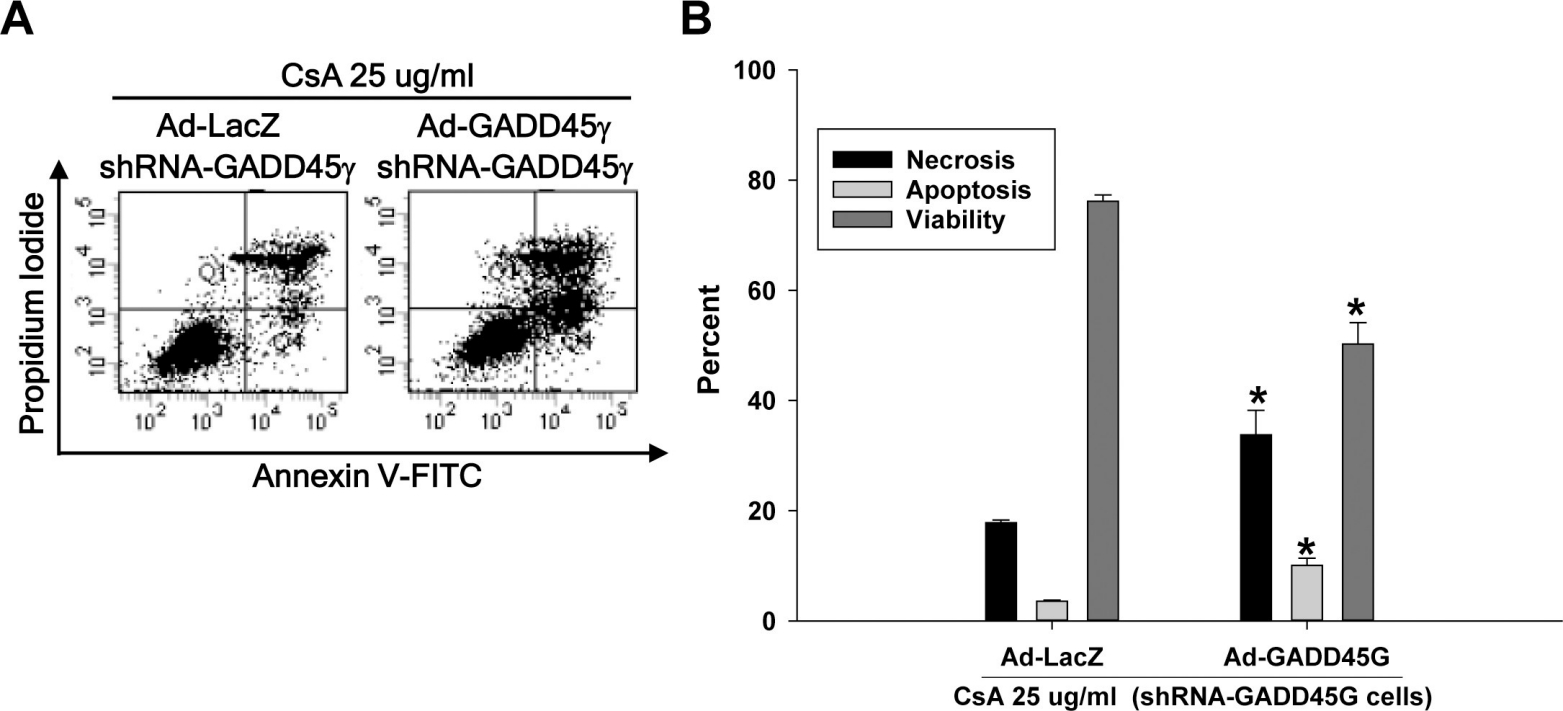
Quantitation of apoptosis and necrosis by flow cytometry in CsA-treated renal tubular cells: The effect of the restoration of GADD45γ expression in GADD45γ knockdown cells. shRNA-GADD45γ cells were infected with 50 MOI of Ad-LacZ or Ad-GADD45γ for 48 hours and then treated with 25 ug/ml CsA for 72 h. **(A)** Representative scatter plots of flow cytometry. Quantitation of **(B)** apoptotic cells (PI negative and Annexin V positive, Q4), **(C)** necrotic cells (PI positive and Annexin V positive, Q2), **(D)** viable cells (PI negative and Annexin V negative, Q3). Each bar represents mean ± SD of 3 experiments. *p < 0.05 vs Ad-LacZ transduced cells (Student's t-test). Ad-GADD45γ(G) represents adenoviral vectors expressing GADD45γ; Ad-LacZ, LacZ adenoviral vectors.

To further clarify the role of GADD45γ in apoptosis and necrosis, HRE cells were incubated with another nephrotoxic agent, CsA, at a concentration of 25 ug/ml, then analyzed by flow cytometry. Like the cisplatin experiment, apoptosis as well as necrosis was significantly decreased in shRNA-GADD45γ HRE cells during the incubation with CsA, resulting in significantly increased survival compared to negative control shRNA-NC HRE cells (Fig 5). Next, GADD45γ knockdown HRE cells were transduced with adenoviral vectors expressing GADD45γ to restore GADD45γ expression, then treated with CsA to evaluate cell death. As expected, the restoration of GADD45γ expression significantly increased apoptosis and necrosis, leading to significantly decreased cell survival compared to LacZ transduced control cells (Fig 6).

### Caspase activation is dependent on GADD45γ expression

**Fig 7:**
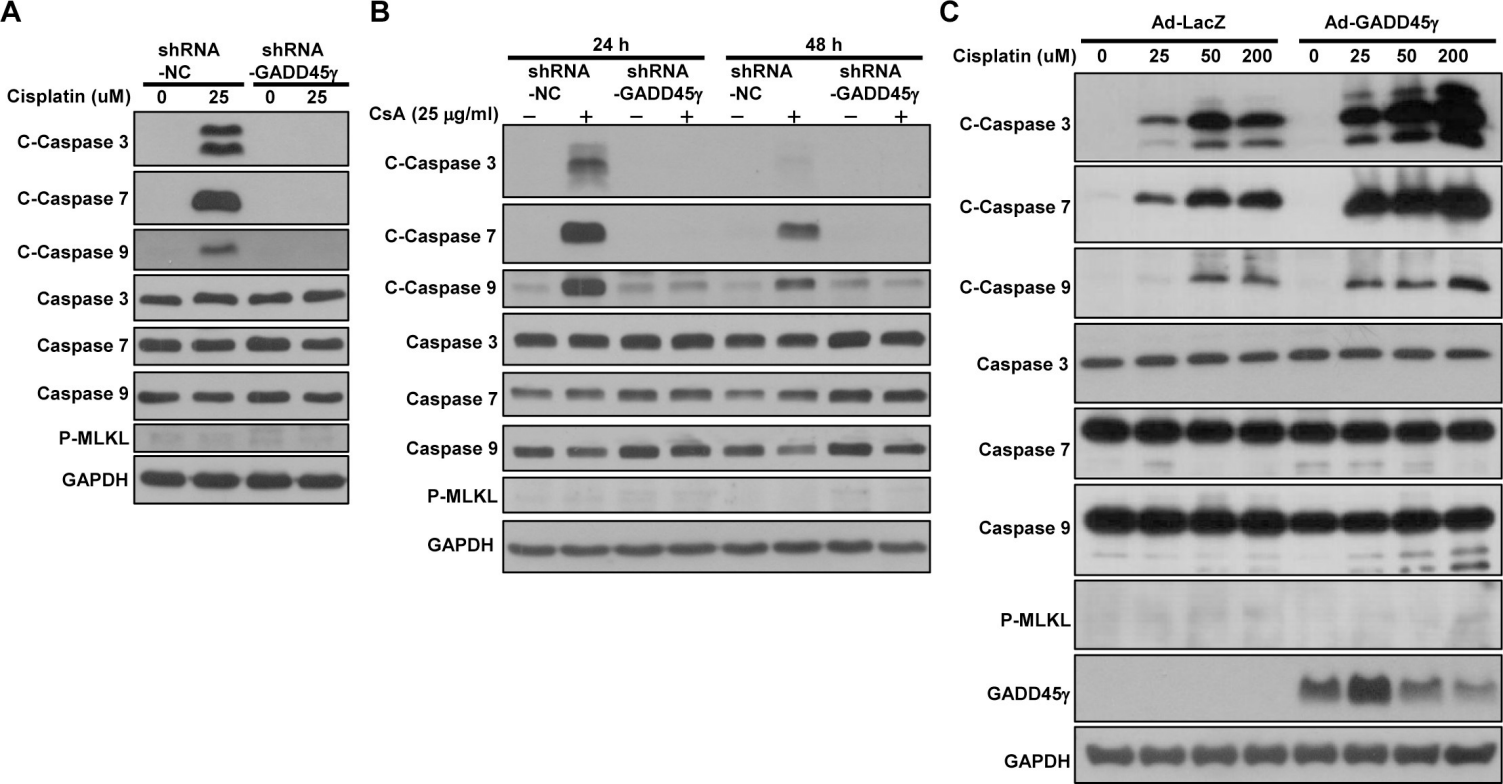
Western blot analysis of caspase activation. **(A)** shRNA-GADD45γ and shRNA-NC HRE cells were incubated with 25 μM cisplatin for 24 hours. **(B)** shRNA-GADD45γ and shRNA-NC HRE cells were incubated with 25 μg/ml CsA for 24–48 hours. **(C)** HRE cells were infected with 50 MOI of adenoviral vectors and then treated with cisplatin at concentrations 25, 50, and 200 μM for 6 hours. Ad-LacZ served as controls. C-caspases represent cleaved (active)-caspases. shRNA-GADD45γ represents shRNA constructs to knockdown GADD45γ; shRNA-NC, shRNA constructs targeting no known genes. Ad-GADD45γ represents adenoviral vectors expressing GADD45γ; Ad-LacZ, LacZ adenoviral vectors.

To gain insight into the mechanism of GADD45γ-mediated regulation of cell death, we decided to examine the expression of caspases, known mediators of apoptosis. MLKL which has recently been implicated as a key mediator of programmed necrosis, termed necroptosis, was also examined [18]. We found that caspases-3 and -7 as well as caspase-9 were activated by cisplatin and CsA as evidenced by the increased cleaved forms, and such activation of caspases was prevented by silencing of GADD45γ (Fig 7A and 7B). Accordingly, the activation of caspases was significantly augmented by GADD45γ overexpression in cisplatin treated cells (Fig 7C), confirming the role of GADD45γ in the activation of caspases. MLKL was not activated by either drug, indicating that necroptosis was not the pathway of cell death.

### Cisplatin-induced cell death is prevented by caspase inhibition

**Fig 8:**
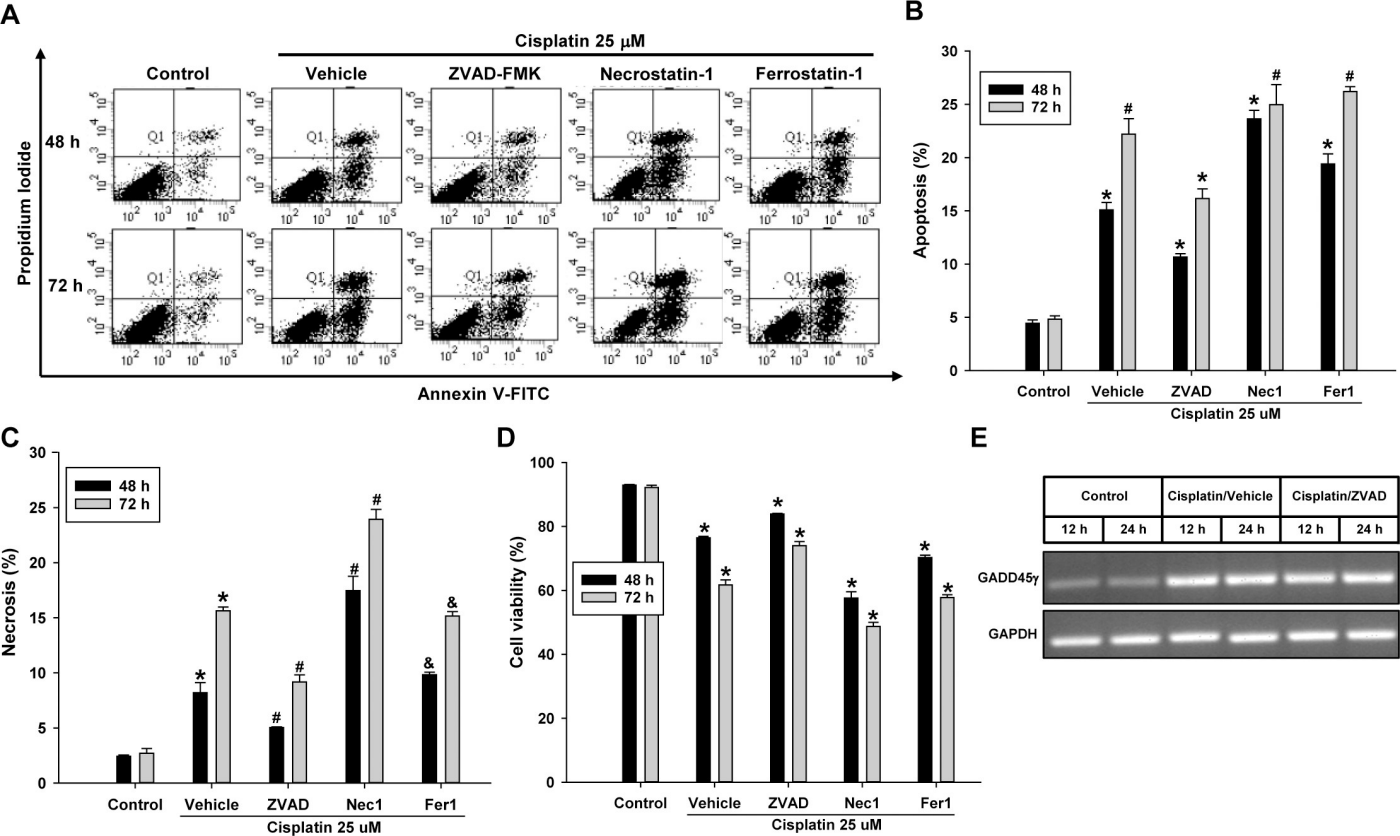
Quantitation of apoptosis and necrosis by flow cytometry in cisplatin-treated renal tubular cells: The effect of caspase inhibition. HRE cells were incubated with 25 μM cisplatin for 48–72 hours with pan-caspase inhibitor ZVAD-FMK, necroptosis inhibitor necrostatin-1, or ferroptosis inhibitor ferrostatin-1. **(A)** Representative scatter plots of flow cytometry. **(B)** Quantitation of apoptotic cells (PI negative and Annexin V positive, Q4). *p<0.05 vs all other corresponding values; #p<0.05 vs control and ZVAD/cisplatin **(C)** Quantitation of necrotic cells (PI positive and Annexin V positive, Q2). *p<0.05 vs all other corresponding values except Fer1/cisplatin (p = ns); #p<0.05 vs all other corresponding values; &p<0.05 vs all other corresponding values except vehicle/cisplatin (p = ns) **(D)** Quantitation of viable cells (PI negative and Annexin V negative, Q3). **(E)** HRE cells were incubated with 25 μM cisplatin for 12–24 hours in the presence or absence of ZVAD-FMK, and the expression of mRNA was evaluated by RT-PCR. *p<0.05 vs all other corresponding values. Each bar represents mean ± SD of 3 experiments. One-way ANOVA with a post-hoc Bonferroni multiple comparison test to compare all groups.

Having found that the activation of caspase is dependent on GADD45γ, we proceeded to determine the role of caspases in renal tubular cell death. To inhibit the activity of caspases, ZVAD-FMK was added to the HRE cells along with cisplatin. For comparison, necroptosis inhibitor necrostatin-1, or ferroptosis inhibitor ferrostatin-1 were added to the cells. Flow cytometry analysis showed that apoptosis induced by cisplatin was significantly decreased by ZVAD-FMK, leading to significantly increased cell survival, which indicates a role of caspases in the induction of apoptosis (Fig 8). Along with apoptosis, necrosis was effectively prevented by ZVAD-FMK as well, indicating that the observed PI/Annexin V double stain should represent a secondary form of necrosis that progressed from apoptosis rather than a primary occurrence of necrotic events (Fig 8). In contrast, primary necrosis inhibitors, necrostatin-1 and ferroptosis, were not effective in preventing apoptosis or necrosis, indicating that non-apoptotic pathways may not contribute to the cisplatin-induced cells death (Fig 8). The absence of the effect of such necrosis inhibitors indicates again that the observed PI/Annexin V double stain should represent secondary necrosis. Additionally, ZVAD-FMK did not change GADD45γ levels in cisplatin treated HRE cells (Fig 8).

### CsA-induced cell death is prevented by caspase inhibition

**Fig 9:**
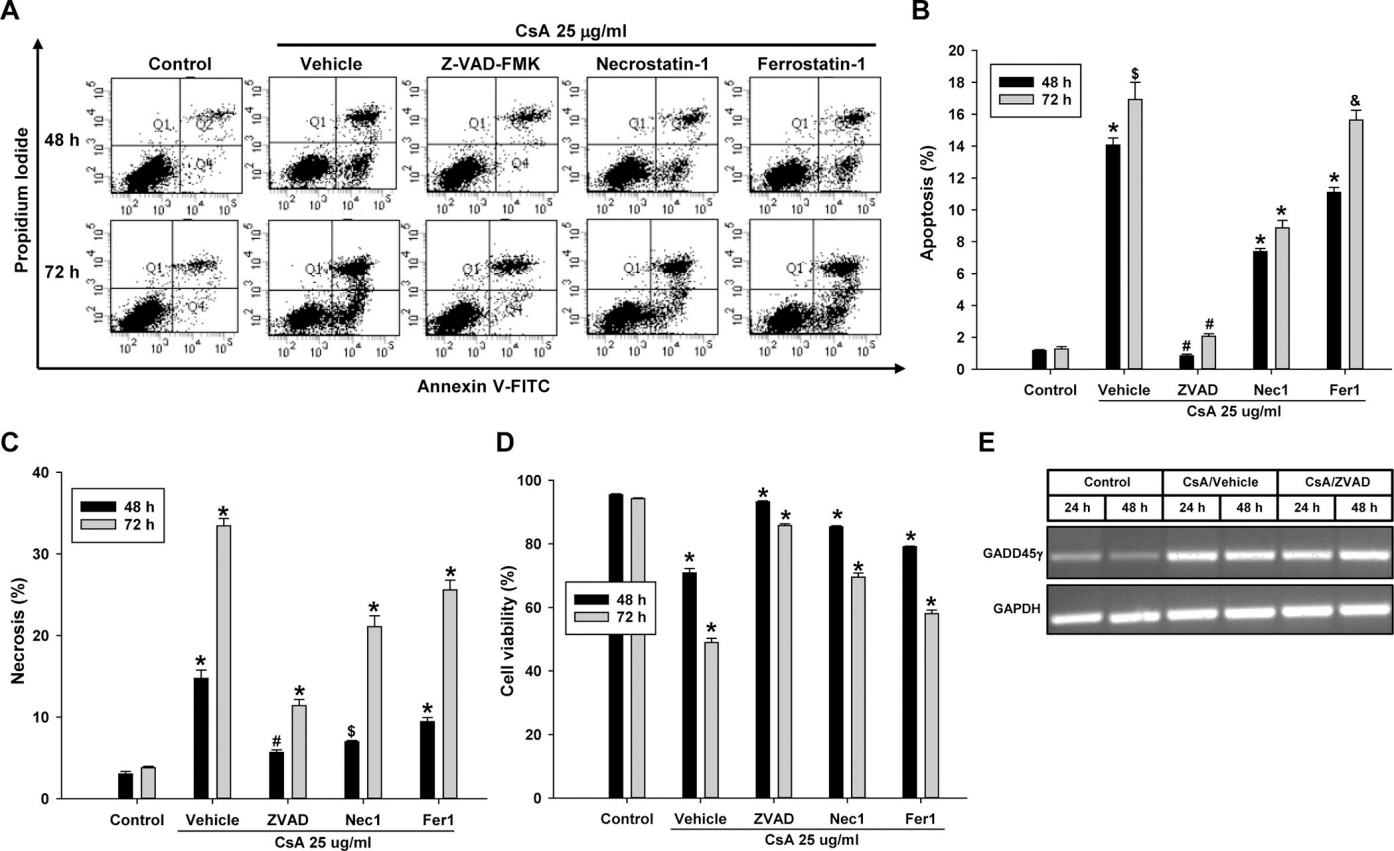
Quantitation of apoptosis and necrosis by flow cytometry in CsA-treated renal tubular cells: The effect of caspase inhibition. HRE cells were incubated with 25 ug/ml CsA for 48–72 hours with pan-caspase inhibitor ZVAD-FMK, necroptosis inhibitor necrostatin-1, or ferroptosis inhibitor ferrostatin-1. **(A)** Representative scatter plots of flow cytometry. **(B)** Quantitation of apoptotic cells (PI negative and Annexin V positive, Q4). *p<0.05 vs all other corresponding values; #p<0.05 vs all other corresponding values except Control (p = ns); $p<0.05 vs all other corresponding values except Fer1/CsA (p = ns); &p<0.05 vs all other corresponding values except Vehicle/CsA (p = ns). **(C)** Quantitation of necrotic cells (PI positive and Annexin V positive, Q2). *p<0.05 vs all other corresponding values; #p<0.05 vs all other corresponding values except Nec1/CsA (p = ns); $p<0.05 vs all other corresponding values except ZVAD/CsA (p = ns). **(D)** Quantitation of viable cells (PI negative and Annexin V negative, Q3). **(E)** HRE cells were incubated with 25 ug/ml CsA for 24–48 hours in the presence or absence of ZVAD-FMK, and the expression of mRNA was evaluated by RT-PCR.*p<0.05 vs all other corresponding values. Each bar represents mean ± SD of 3 experiments. One-way ANOVA with a post-hoc Bonferroni multiple comparison test to compare all groups.

To elucidate the role of caspases in CsA-induced cell death, ZVAD-FMK was added to the HRE cells along with CsA. For comparison, the effect of necrostatin-1 and ferrostatin-1 were evaluated as well. Flow cytometry analysis showed that ZVAD-FMK significantly decreased apoptosis induced by CsA, indicating a role of caspases in apoptosis. ZVAD-FMK was also effective in preventing secondary necrosis, as in the cisplatin experiment. Though ZVAD-FMK was most effective for prevention of cell death, necrostatin-1 and ferrostatin-1 were also shown to be effective (Fig 9), indicating that although caspase-dependent apoptosis is mainly responsible for cell death, non-apoptotic pathways may play a role in CsA-induced renal tubular cells death. Additionally, ZVAD-FMK did not change GADD45γ levels in CsA treated HRE cells (Fig 9).

## Discussion

In this study, we sought to determine the role of GADD45γ in the death pathways of human renal tubular cells induced by the nephrotoxic drugs cisplatin and CsA which are known to cause renal tubular cell injury and related apoptosis. We found that the expression of GADD45γ gene was upregulated upon treatment with cisplatin and CsA, and that the inhibition of GADD45γ significantly reduced the apoptotic as well as necrotic cell death induced by the drugs. Accordingly, the overexpression of GADD45γ significantly augmented both modes of cell death induced by these drugs. The major executioner caspases, caspase-3 and caspase-7 and the initiator caspase, caspase-9 [19], were all activated by cisplatin and CsA, and such activation was significantly inhibited by GADD45γ knockdown, or significantly augmented by GADD45γ overexpression, indicating that the activation of caspases is dependent on the expression of GADD45γ. ZVAD-FMK, the pan-caspase inhibitor, effectively prevented apoptosis induced by these drugs, confirming the role of caspases in apoptotic cell death. The finding that necrosis (PI/Annexin V double stain) was more effectively prevented by ZVAD-FMK than the inhibitors of primary regulated necrosis, necrostatin-1 and ferrostatin-1, suggests that the observed necrosis should be a secondary event resulting from the progression of the apoptotic program towards necrosis [20]. In addition, phospho-MLKL was not detected during the induction of cell death, indicating that necroptosis [18, 21, 22] was neither induced by cisplatin and CsA, nor affected by GADD45γ expression. Together, this set of evidence proves that GADD45γ is required for caspase activation and subsequent caspase-dependent apoptosis of renal tubular cell induced by nephrotoxic drugs.

To date, no data exists which implicates GADD45γ in apoptotic tubular cell death in AKI inflicted by direct renal insults such as renal ischemia [6, 7], sepsis [8] and nephrotoxins [9–13]. Of these insults, nephrotoxin is the most appropriate for *in vitro* induction of apoptosis, thus we selected cisplatin and CsA, well-known nephrotoxic drugs for our experiment. In fact, apoptosis is known to be a critical mode of renal tubular cell death induced by cisplatin, especially at low doses [23], where caspase activation is the key step [24]. It has been demonstrated that p53 plays a critical role in apoptosis by induction of PUMA (p53 upregulated modulator of apoptosis), p53-responsive pro-apoptotic Bcl-2 family protein [25, 26], or PIDD (p53-induced death domain protein) [27]. PIDD is involved in the mechanism of cisplatin-induced caspase-2 activation with subsequent mitochondrial release of AIF (apoptosis-inducing factor) causing apoptosis [27]. Others have shown that cisplatin activates cdk2 which, in turn, causes E2F1 activation, resulting in apoptosis of renal tubular cells [28]. CsA, another nephrotoxic drug, has also been shown to cause apoptotic cell death of renal tubular cell, and that such apoptosis is mediated by caspases [29]. The expression of specific apoptotic genes, such as p53, Bax and Fas ligand were shown to be enhanced in response to CsA in the kidney [30]. Above all, the mitochondrial pathway seems to be most critical in CsA-induced apoptosis, and it has been shown that CsA promotes the translocation of Bax to the mitochondria leading to release of cytochrome C with subsequent activation of caspase-3 and caspase-9, resulting in apoptosis of renal tubular cells [31].

GADD45γ has been shown to induce apoptosis in some types of cells, though no such data exists in renal tubular cells. In cancer cells and myocytes, evidence suggests that the induction of apoptosis by GADD45γ is dependent on p38 mitogen activated protein kinase (MAPK) or c-Jun N-terminal kinase (JNK) MAPK signaling pathways [2, 32], and that GADD45γ activates MAPK pathways via signaling cascades involving MTK1/ MEKK4 in response to environmental stresses [4, 33, 34].

We reported that GADD45γ promotes renal fibrosis in unilateral ureteral obstruction (UUO), a model mimicking chronic renal injury [4], and is associated with progression of IgA nephropathy [5], suggesting that GADD45γ may promote progression of CKD. Previous data demonstrated that the progression of CKD is the result of caspase-dependent apoptosis and subsequent atrophy [35–37], thus together with our current data, it may be hypothesized that heightened expression of GADD45γ may contribute to the progression of CKD through the induction of renal tubular cell apoptosis.

Cell death pathways may be stimulus-specific; hence we do not know whether our findings can be extended to other types of nephrotoxic insults. Given the tight link between GADD45γ and caspases shown in this study, however, it is possible that renal tubular cell death, if it is caspase dependent, may be regulated by GADD45γ expression irrespective of the type of stimulus.

In conclusion, we propose a novel pathway of apoptosis of renal tubular cells where GADD45γ plays a critical role. We hope our findings contribute to the knowledge of the mechanisms of tubular cell death in AKI. Knowledge of these mechanisms and determinants of cell death may reveal new therapeutic targets for the mitigation of AKI in the future.
